# Real-Time Robust Path Following of a Biomimetic Robotic Dolphin in Disturbance-Rich Underwater Environments

**DOI:** 10.3390/biomimetics10100687

**Published:** 2025-10-13

**Authors:** Yukai Feng, Sijie Li, Zhengxing Wu, Junzhi Yu, Min Tan

**Affiliations:** 1 School of Artificial Intelligence, University of Chinese Academy of Sciences, Beijing 100049, China; fengyukai2021@ia.ac.cn (Y.F.); lisijie2020@ia.ac.cn (S.L.); min.tan@ia.ac.cn (M.T.); 2 The Key Laboratory of Cognition and Decision Intelligence for Complex Systems, Institute of Automation, Chinese Academy of Sciences, Beijing 100190, China; 3 The State Key Laboratory for Turbulence and Complex Systems, Department of Advanced Manufacturing and Robotics, College of Engineering, Peking University, Beijing 100871, China; junzhi.yu@ia.ac.cn

**Keywords:** robotic dolphin, underwater bionic robot, path-following control

## Abstract

In ocean engineering, path following serves as a fundamental capability for autonomous underwater vehicles (AUVs), enabling essential operations such as environmental exploration and inspection. However, for robotic dolphins employing dorsoventral undulatory propulsion, the periodic pitching induces strong coupling between propulsion and attitude, posing significant challenges for precise path following in disturbed environments. In this paper, a real-time robust path-following control framework is proposed for robotic dolphins to address these challenges. First, a novel robotic dolphin platform is presented by integrating a dorsoventral propulsion mechanism with a passive peduncle joint, followed by the systematic formulation of a full-state dynamic model. Then, a minimum-snap-based path optimizer is constructed to generate smooth and dynamically feasible trajectories, improving path quality and motion safety. Subsequently, a robust model predictive controller is developed, which incorporates control surface dynamics, a nonlinear disturbance observer, and a Sigmoid-based disturbance-grading mechanism to ensure fast attitude response and precise tracking performance. Finally, extensive simulations under various environmental disturbances validate the effectiveness of the proposed approach in both trajectory optimization and robust path following. The proposed framework not only demonstrates strong robustness in path following and disturbance rejection, but also provides practical guidance for future underwater missions such as long-term environmental monitoring, inspection, and rescue.

## 1. Introduction

With the advancement of ocean engineering technologies, tasks such as resource exploration, environmental monitoring, and infrastructure inspection are imposing increasingly stringent requirements on the intelligence and maneuverability of autonomous underwater vehicles (AUVs) [1,2,3,4]. Although conventional AUVs equipped with propulsion systems exhibit considerable endurance and payload capacity, they are often constrained in terms of maneuverability, environmental adaptability, and energy efficiency [5,6,7]. Inspired by the outstanding swimming capabilities of fish, biomimetic robotic fish have attracted considerable attention for their low disturbance, high propulsion efficiency, and strong adaptability in aquatic environments [8,9]. Among these, biomimetic robotic dolphins driven by dorsoventral undulatory propulsion achieve distinctive integration of high-speed cruising and agile maneuverability [10,11,12], rendering them highly suitable for long-range autonomous operations, such as target search, ecological monitoring, and rescue missions.

In contrast to conventional underwater vehicles that typically decouple propulsion and attitude control, robotic dolphins achieve functional unification through their distinctive biomimetic mechanical design. By coupling thrust generation with body dynamics in a dolphin-inspired manner, the design provides multiple advantages, including the ability to execute highly agile maneuvers along complex trajectories, improved adaptability to varying environmental conditions, and increased resilience to external disturbances. Consequently, robotic dolphins demonstrate reliable performance in dynamic and unstructured underwater environments, supporting applications such as environmental monitoring, infrastructure inspection, and cooperative multi-robot operations [13]. To meet these operational demands, various motion planning and control strategies have been explored for robotic dolphins. Wu et al. investigated the role of a controllable fluke in regulating the gliding motion of a robotic dolphin, and proposed a pitch control strategy based on dynamic modeling and experimental validation to enhance underwater gliding performance [14]. Li et al. developed a dolphin-inspired underwater vehicle with an intermittent-propulsion mode, enabling energy-efficient long-range navigation for marine exploration [15]. Notably, for complex tasks such as autonomous cruising, obstacle avoidance, and terrain following, high-precision and robust path following is a fundamental capability for ensuring the efficiency and reliability of underwater operations.

In recent years, extensive research has focused on improving the accuracy and robustness of underwater path following for autonomous underwater vehicles. Existing approaches are typically categorized into three categories: geometric, control-theoretic, and learning- or perception-driven methods [16,17,18]. Representative methods from each category have been proposed to address the challenges of dynamic and uncertain underwater environments. Du et al. introduced a geometry-based indirect adaptive line-of-sight (LOS) guidance framework, which integrates over-parameterized disturbance estimation to achieve robust AUV path following under strong time-varying ocean currents [19]. Zhou et al. developed a model predictive control (MPC)-based path-following method for underactuated AUVs, combining hydrodynamic parameter identification and adaptive line-of-sight guidance for robust and constrained control [20]. Ma et al. developed a ModelPPO algorithm that integrates neural dynamics into a proximal policy optimization algorithm for robust and sample-efficient 3-D path-following control in dynamic underwater environments [21]. These approaches have been widely applied to conventional AUVs and have demonstrated effectiveness in mitigating environmental disturbances and tracking complex trajectories [22,23,24]. However, existing control methods are typically developed based on the assumption of stable heading and attitude dynamics, which limits their applicability to robotic dolphins that employ biomimetic propulsion with flexible actuation and nonlinear motion characteristics. These platforms often exhibit periodic surge–pitch coupling and oscillatory behavior, thereby undermining the effectiveness of traditional control approaches. Consequently, achieving high-precision and robust path following for such platforms remains a critical and underexplored challenge.

Specifically, biomimetic robotic dolphins employing dorsoventral undulatory propulsion generate thrust through periodic oscillations along the dorsoventral axis. This distinctive mechanism inherently induces significant periodic pitching, resulting in strong coupling between attitude and propulsion direction. While offering excellent maneuverability and propulsion efficiency, this mechanism poses considerable challenges for path-following control. On one hand, the physical constraints imposed by undulatory propulsion limit the ability of the robotic dolphin to adjust its attitude, particularly in the pitch direction. As a result, when following paths with sharp transitions or high-curvature segments, the robot tends to exhibit significant vertical oscillations and pitch changes, which adversely affect motion smoothness, reduce propulsion efficiency, and significantly increase control complexity. On the other hand, as an underactuated platform, the biomimetic robotic dolphin must perform tasks under conditions in which attitude dynamics have a notable impact on the propulsion direction. Meanwhile, the system is subject to various disturbances, such as environmental perturbations and hydrodynamic forces, which place stringent demands on both the predictive accuracy and robustness of the controller. Recent advances in the field of underwater robotics have produced a variety of approaches to improve autonomy and robustness under complex environmental conditions. For underactuated AUVs, numerous robust path-following controllers have been developed to mitigate the effects of current disturbances, enabling reliable long-duration navigation in uncertain flow fields [25]. In addition, adaptive disturbance observer-based schemes have been widely investigated for trajectory tracking, where online estimation and compensation for environmental disturbances reduce reliance on conservative disturbance bounds and improve control efficiency [26]. Beyond conventional AUV platforms, biomimetic robotic fish have emerged as a growing research focus. Leveraging undulatory propulsion mechanisms, these prototypes demonstrate superior maneuverability and energy efficiency compared to traditional propeller-driven vehicles. Recent experimental studies have further validated their ability to execute coordinated motion and disturbance rejection strategies in constrained aquatic environments, thereby expanding the application scope of bio-inspired underwater robots [27]. Despite these promising developments, few studies have systematically integrated curvature-constrained minimum-snap path planning, graded robust MPC, and adaptive control allocation tailored to the unique dynamics of biomimetic propulsion. Therefore, it is necessary to develop a path optimization strategy tailored to the propulsion and maneuvering characteristics of the biomimetic robotic dolphin, ensuring both smoothness and reachability. In addition, a path-following controller with high adaptability and robustness should be proposed to guarantee the stability and safety of the tracking process. The coordinated integration of path optimization and control is critical for achieving reliable and stable operation in highly disturbed environments.

This paper presents a systematic investigation into path optimization and robust path following for biomimetic robotic dolphins, and proposes an integrated framework that addresses the challenges in path following of such platforms. The main contributions of this work are summarized as follows:

1. A novel biomimetic robotic dolphin is developed by integrating a dorsoventral propulsion mechanism with a passive compliant peduncle joint, enabling high-frequency, symmetric dorsoventral oscillations that ensure both maneuverability and stability during locomotion.
2. A path optimization strategy based on a curvature-constrained minimum leap distance algorithm is proposed, which generates a smooth and safe reference path aligned with the maneuvering capabilities of the robotic dolphin. By mitigating frequent vertical oscillations and abrupt pitch transitions, the proposed method improves path tracking performance and enhances the overall safety of the motion.
3. A robust model predictive controller (RMPC) is developed to handle path following under diverse disturbances by incorporating the dynamic response of control surfaces and integrating a nonlinear disturbance observer with a Sigmoid-based grading mechanism. This design enables adaptive regulation of input margins and real-time control allocation, improving dynamic responsiveness and enhancing disturbance rejection performance during path following.
4. A series of simulation experiments under various environmental disturbance are conducted to validate the effectiveness of the proposed approach in both paths optimization and robust control.

The remainder of this paper is organized as follows. Section 2 describes the mechanical design and dynamic model of the robotic dolphin. Section 3 details the proposed path optimization strategy and robust path-following controller. Section 4 presents the simulation results and performance analysis. Finally, Section 5 concludes the paper and discusses potential directions for future work.

## 2. System Design and Modeling of the Bionic Robotic Dolphin

In this section, a novel robotic dolphin system is introduced, featuring redundant control surfaces and a passive peduncle joint to enhance maneuverability and adaptability. Furthermore, a full-state dynamic model is developed based on the Newton–Euler formulation, integrated with a quasi-steady lift–drag force model to accurately capture both rigid-body dynamics and hydrodynamic effects.

### 2.1. Overview of Bionic Robotic Dolphin System

**Figure 1:**
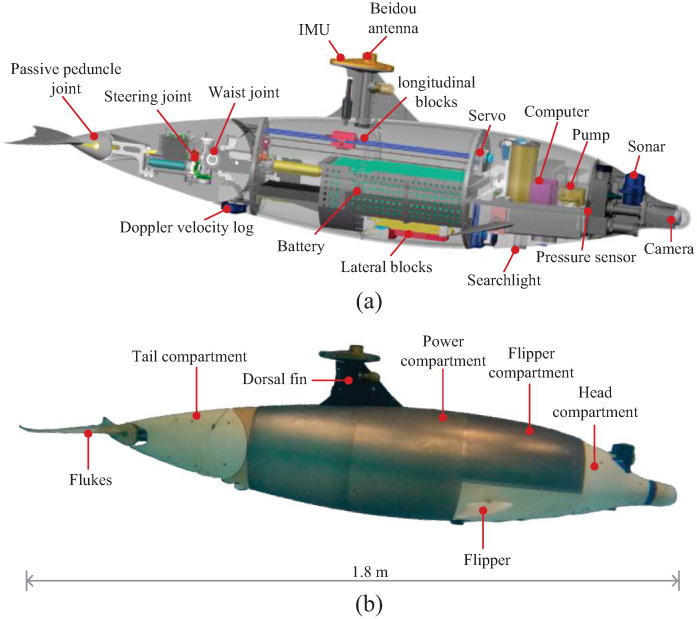
The developed bionic robotic dolphin. (**a**) Mechanical structure; (**b**) prototype.

Inspired by the bottlenose dolphin, a novel biomimetic robotic dolphin is developed, featuring a body design that replicates the morphological proportions of an adult specimen. As shown in Figure 1, the platform employs a dorsoventral undulatory propulsion mechanism that integrates a waist joint, a steering joint, and a passive peduncle joint, enabling high-speed and biomimetic locomotion. To improve maneuverability and enhance control robustness, the system incorporates multiple redundant actuation modules, including a net buoyancy regulation unit, a center-of-mass shifting mechanism, and a vertical rudder. The robotic dolphin measures 1.8 m in length, 0.34 m in width, and 0.48 m in height, with a total mass of approximately 63 kg.

The dorsoventral undulatory propulsion mechanism facilitates biomimetic motion that closely emulates the swimming patterns of real dolphins. Unlike previous designs [11], the propulsion system of the robotic dolphin comprises a waist-pitch joint, a yaw-pitch joint, and a passive peduncle joint. The waist-pitch joint serves as the primary actuator, generating thrust through dorsoventral oscillations. The addition of the yaw-pitch joint improves maneuverability and directional control during turning motions. Meanwhile, the passive peduncle joint, equipped with a spring-based restoring mechanism, effectively attenuates surge-induced pitching, thereby improving motion stability.

Moreover, to maintain stable posture in disturbed environments, the robotic dolphin incorporates multiple attitude control mechanisms, including a flipper system, a buoyancy regulation unit, and a movable center-of-mass module. Specifically, the flipper system provides pitch and yaw control by adjusting the deflection angles of the fin surfaces. The buoyancy regulation unit, located in the head compartment, uses a peristaltic pump and water reservoir to adjust net buoyancy, enabling control of vertical position and orientation. The center-of-mass regulation module comprises longitudinal and lateral sliding blocks, in which the former enables pitch angle control and the latter enhances roll stability and overall control effectiveness.

### 2.2. Coordinate Frames and Variable Definitions

**Figure 2:**
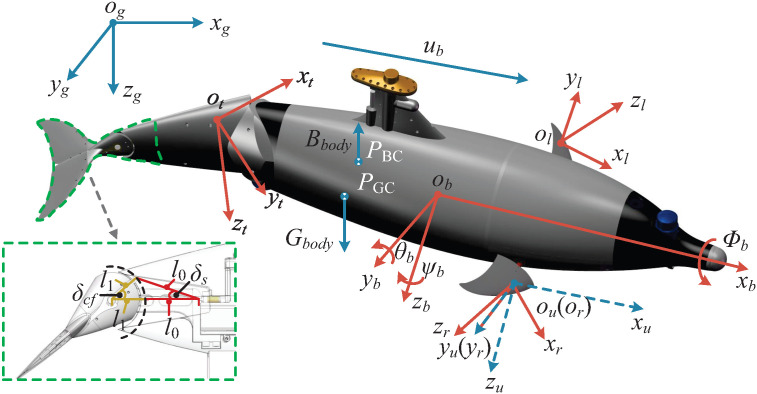
The coordinate frames of the robotic dolphin.

As shown in Figure 2, to construct the dynamic model of the biomimetic robotic dolphin, multiple coordinate frames aligned with the right-hand rule are defined, including the global inertial frame $o_{g}-x_{g}y_{g}z_{g}$, the body-fixed frame $o_{b}-x_{b}y_{b}z_{b}$, and the waist joint frame $o_{t}-x_{t}y_{t}z_{t}$. In addition, to describe the motion of the fin surfaces, fin-fixed coordinate frames $o_{i}-x_{i}y_{i}z_{i}$ are introduced, where i=l,r,c denotes the left flipper, right flipper, and flukes. Additionally, a hydrodynamic frame $o_{u}-x_{u}y_{u}z_{u}$ is also defined, whose origin can be aligned with any of the aforementioned frames, and is used for computing hydrodynamic forces on the fin surfaces. To ensure stable attitude regulation, the center of buoyancy $P_{BC}$ is positioned above the center of gravity $P_{GC}$. The magnitudes of buoyancy $B_{body}$ and gravitational force $G_{body}$ are denoted accordingly. Throughout this study, all vectors and matrices are denoted with a superscript indicating their associated coordinate frame. For example, the rotation matrix Rbg represents the orientation transformation from the body-fixed frame to the global inertial frame. The cross product ω×r is represented using the skew-symmetric matrix form ${\left[\omega \right]}_{\times }r$.

The three-dimensional position and orientation of the robotic dolphin are governed by the following kinematic equations:

(1) P˙bg=RbgVbbR˙bg=[Ωbg]×Rbg=Rbg[Ωbb]×,

where Pbb denotes the position vector in the inertial frame, and Vbb, Ωbb represent the linear and angular velocities in the body frame.

### 2.3. Dynamic Modeling of the Robotic Dolphin

To characterize the dynamic behavior of the biomimetic robotic dolphin and establish a foundation for controller design, a comprehensive three-dimensional dynamic model is developed based on the Newton–Euler formulation. The model accounts for hydrodynamic forces acting on each body segment, the net buoyant force generated by the pumping system, and generalized forces resulting from the movable mass blocks. The detailed mathematical formulation is presented below.

(1) Hydrodynamic modeling

The hydrodynamic forces acting on each body segment are computed as follows:

(2) Fiv=0.5ρSiVii2−Ci,d(αi),Ci,sf(βi),−Ci,l(αi)T

(3) τiv=0.5ρSiVii2Ci,τx(βi),Ci,τy(αi),Ci,τz(βi)T+kiΩiv

(4) αi=tan−1Vi,ziVi,xi

(5) βi=sin−1Vi,yiVii,

where Fiv and τiv denote the hydrodynamic force and moment acting on segment *i*, respectively. $\alpha_{i}$ denotes the angle of attack associated with the *i*-th body segment, where *i* corresponds to the main body, flukes, or flipper of the robotic dolphin. Vii is the velocity vector of the *i*-th body segment, and $\beta_{i}$ represents the associated sideslip angle. ρ represents the fluid density, and $S_{i}$ is the reference surface area of the segment *i*. The coefficients $C_{i,d}\left(\alpha_{i}\right)$, $C_{i,sf}\left(\beta_{i}\right)$, $C_{i,l}\left(\alpha_{i}\right)$, $C_{i,\tau_{x}}\left(\beta_{i}\right)$, $C_{i,\tau_{y}}\left(\alpha_{i}\right)$, and $C_{i,\tau_{z}}(\beta_{i})$ quantify the hydrodynamic characteristics of each segment. $k_{i}$ denotes the rotational damping coefficient, and Ωiv indicates its angular velocity in the hydrodynamic frame.

For tail fins with compliant mechanisms, the associated hydrodynamic forces exhibit pronounced nonlinear behavior, thereby necessitating a modified model for accurate computation:

(6) $$\begin{array}{cc} \alpha_{cf} & =\alpha_{cf,0}+\delta_{cf} \end{array}$$

(7) $$\begin{array}{cc} \beta_{cf} & =\beta_{cf,0}, \end{array}$$

where $\alpha_{cf}$ and $\alpha_{cf,0}$ denote the attack angles of the flukes and peduncle, respectively. In this context, $\delta_{cf}$ denotes the caudal fin deflection angle, representing the elastic deformation of the compliant fin structure, which directly modulates the thrust generated during oscillatory motion. $\beta_{cf,0}$ denotes the sideslip angle of the peduncle. Due to the correspondence between spring deformation and fin deflection, $\delta_{cf}$ can be treated as a system state and iteratively updated during the computation process.

As shown in Figure 2, $\delta_{cf}$ is computed using a second-order dynamic model that incorporates the interaction between spring-induced torque and hydrodynamic forces. The governing equation is given as follows:

(8) ∥Tsany∥+0.5ρScf∥Vcft∥2Ccf,τy(αcf)=Jcft,yδ¨cf

(9) ∥Tsany∥=ks(l0+l1)2+l12−2(l0+l1)l1cosδcf−l0·(l0+l1)sinδs

(10) $$\begin{array}{cc} \; & \delta_{s}=\cos^{-1}\;\frac{{\left(l_{0}+l_{1}\right)}^{2}-\left(l_{0}+l_{1}\right)l_{1}\cos\delta_{cf}}{\left(l_{0}+l_{1}\right)\sqrt{{\left(l_{0}+l_{1}\right)}^{2}+l_{1}^{2}-2\left(l_{0}+l_{1}\right)l_{1}\cos\delta_{cf}}}, \end{array}$$

where |Tsany|| denotes the spring-generated torque, and $k_{s}$ is the stiffness coefficient of the spring. $l_{0}$, $l_{1}$, and $\delta_{s}$ represent the geometric parameters of the structural components shown in Figure 2. $\delta_{cf}$ is the deflection angle of the flukes, and $J_{cft,y}$ represents the moment of inertia.

(2) Buoyancy modeling

Considering the buoyancy variation induced by pump actuation, the corresponding force and moment are expressed as follows:

(11) Fbuoyancyb=ρgSsyringehRbTg00−1T

(12) τbuoyancyb=ρgSsyringehrb,pumpb×RbTg00−1T,

where $S_{syringe}$ denotes the cross-sectional area of the pump piston, and *h* is the piston displacement. The vector rb,pumpb represents the position from the origin of the body frame to the point of force application.

(3) Gravity modeling of movable mass blocks

The additional force and moment generated by the longitudinal and lateral sliding blocks for center-of-mass control are expressed as

(13) Fmassb=(mlg+mlt)gRbTg001T

(14) τmassb=(mlgrb,mlgb×+mltrb,mltb×)gRbTg001T,

where $m_{lg}$ and $m_{lt}$ represent the masses of the longitudinal and lateral sliding blocks, respectively. The vectors rb,mlgb and rb,mltb denote the corresponding position in the body frame.

(4) Full-state dynamic modeling

Substituting the above external force components into the Newton–Euler equations results in the acceleration of the robotic dolphin. Accordingly, the full-state dynamic model of the system is formulated as

(15) V˙bbΩ˙bb=−P−1HVbbΩbb+P−1FextbTextbP=mbI3×3−mbrb,cbb×mbrb,cbb×JbH=mbΩbb×−mbΩbb×rb,cbb×mbrb,cbb×Ωbb×Ωbb×Jb,

where V˙bb and Ω˙bb denote the linear and angular acceleration of the robotic dolphin in the body frame. P denotes the mass matrix, which includes the total mass, moments of inertia, and center-of-mass distribution of the system. *H* represents the Coriolis and centripetal coupling terms. Fextb and Textb are the resultant external force and moment vectors acting on the system. Moreover, $m_{b}$, Jb, and rb,cbb denote the total mass, the inertia tensor, and the position vector from the body-fixed frame origin to the center of mass.

## 3. Path Optimization and Robust Following Control

**Figure 3:**
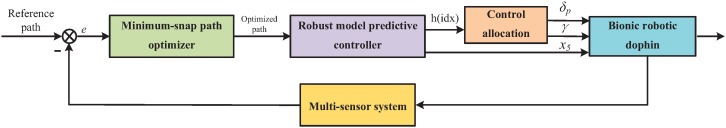
The path-following control framework for the robotic dolphin.

In this section, the proposed robust path-following framework is detailed, as illustrated in Figure 3. The framework consists of a minimum-snap path optimizer, a robust model predictive controller, a nonlinear disturbance observer, and a control allocation module.

### 3.1. Path Optimization for the Robotic Dolphin

Given the limited pitch maneuverability of the robotic dolphin, the reference path is required to minimize vertical oscillations and sudden pitch variations to ensure safety. To address this, a minimum-snap path optimizer is designed to generate smooth and dynamically feasible paths.

Specifically, the maximum pitch curvature $\kappa_{\max}$ and the path point curvature $\kappa_{i}$ are initially estimated based on the current swimming speed of the robotic dolphin:

(16) $$\kappa_{\max}=\frac{∥V_{b}∥}{L}$$

(17) $$\kappa_{i}=\frac{\left|{\dot{x}}_{i}{\ddot{z}}_{i}-{\dot{z}}_{i}{\ddot{x}}_{i}\right|}{{\left({\dot{x}}_{i}^{2}+{\dot{z}}_{i}^{2}\right)}^{3/2}},$$

where $∥V_{b}∥$ is the resultant velocity in the vertical plane, and *L* is the minimum turning radius during pitch maneuvers. Based on these estimates, waypoints that meet pitch constraints are selected to ensure the generated trajectory remains within the feasible control range. Consequently, the minimum-snap path optimization problem is reformulated in a compact quadratic programming (QP) form as follows:

(18) $$\left\{\begin{array}{cc} \min_{\vec{p}} & {\vec{p}}^{T}Q\vec{p}\\ s.t. & A\vec{p}=\vec{d}\\ & C_{j}{\vec{p}}_{j}=C_{j+1}{\vec{p}}_{j+1},\;\forall j=1,\ldots ,M-1, \end{array}\right.$$

where $\vec{p}={\left[\begin{array}{ccc} {\vec{p}}_{1}^{T} & \ldots & {\vec{p}}_{M}^{T} \end{array}\right]}^{T}$ is the stacked polynomial coefficient vector of all *M* trajectory segments, Q is a block-diagonal Hessian matrix depending on time durations $T_{i}$ and snap order, and A and $\vec{d}$ encode the initial and terminal conditions. $C_{j}$ denotes the continuity constraint matrices enforcing equality of derivatives between adjacent segments *j* and j+1.

### 3.2. Robust Model Predictive Controller Design

To address the impact of fluid disturbances encountered in dynamic aquatic environments, the hydrodynamic forces generated by fin deflections are abstracted as virtual control inputs, decoupled from physical actuator commands. This decoupling enables adaptive adjustment of the control input mapping under varying flow conditions. Based on the full-state dynamic model of the robotic dolphin given in (15), a set of nonlinear differential equations is formulated to support path-following control:

(19) x˙1=∥Vb∥cos(x3−α)x˙2=−∥Vb∥x3+α∥Vb∥+δ2(x→)x˙3=x4+δ3(x→)x˙4=−λ1x4+λ2x3+λ3x5+u1+δ4(x→)x˙5=−1TΔrx5+1TΔruΔrlmx+δ5(x→).

Here, $x_{1}$ and $x_{2}$ represent the horizontal and vertical displacements in the global frame. $x_{3}$ is the pitch angle, and $x_{4}$ is the angular velocity. $x_{5}$ represents the displacement of the longitudinal slider, and α denotes the flow angle of attack. The coefficients $\lambda_{i}$ (i=1,2,3) are hydrodynamic parameters that characterize the coupling between the dolphin’s body motion and the surrounding fluid, determined by its morphology, mass distribution, and displacement volume, and they directly influence the restoring and damping effects in the dynamic response. $u_{\Delta r\mathrm{lmx}}$ denotes the desired slider offset, and $\delta_{i}\left(\vec{x}\right)$ (i=1,2,3) represent model approximation errors.

To enable the implementation of the MPC algorithm, the continuous-time system is discretized into the following form:

(20) $$\begin{array}{c} x\left(k+1\right)=A_{d}x\left(k\right)+B_{d}u\left(k\right)+w\left(k\right), \end{array}$$

where $w\left(k\right)={\left[\delta_{2}\left(k\right),\;\delta_{3}\left(k\right),\;\delta_{4}\left(k\right),\;\delta_{5}\left(k\right)\right]}^{T}$ denotes a bounded disturbance vector. The discrete-time matrices are defined as $A_{d}^{d}=e^{A\Delta T}$ and $B_{d}=e^{A\Delta T}\int_{0}^{T}e^{-A\tau }d\tau B$, where ΔT is the sampling period.

The path-following control problem of the biomimetic robotic dolphin is thus modeled as a linear discrete-time state-space system with disturbances. Accordingly, the path-tracking error dynamics model can be formulated as

(21) $$\begin{array}{cc} e(k+1) & =r(k+1)-x(k+1)\\ & =A_{d}e\left(k\right)+{\bar{B}}_{d}u\left(k\right)+\xi \left(k\right)-w\left(k\right), \end{array}$$

where e(k)=r(k)−x(k) denotes the tracking error between the reference and actual states, and r(k+1)=r(k)+Δr(k) defines the reference update. The term ξ(k) represents equivalent disturbances induced by path dynamics and model uncertainties. Additionally, ${\bar{B}}_{d}=-B_{d}$.

To enhance system robustness and mitigate the influence of disturbances, a robust model predictive controller is proposed. The corresponding nominal system model is formulated as

(22) $$\begin{array}{c} z\left(k+1\right)=A_{d}z\left(k\right)+{\bar{B}}_{d}v\left(k\right), \end{array}$$

where $z\left(0\right)=e\left(0\right)$ represents the initial tracking error state. The corresponding cost function is defined as

(23) $$\begin{array}{cc} J(k)= & \sum_{n=1}^{N-1}\left({∥z(n|k)∥}_{Q}^{2}+{∥v(n-1|k)∥}_{R}^{2}\right)\\ & +{∥z(N|k)∥}_{P}^{2}+{∥v(N-1|k)∥}_{R}^{2}, \end{array}$$

where *Q* and *R* are the weighting matrices for the predicted state and the control input. *P* is the terminal cost matrix, which satisfies the following discrete-time Lyapunov equation:

(24) $$\begin{array}{c} P-{\left(A_{d}-{\bar{B}}_{d}K\right)}^{T}P\left(A_{d}-{\bar{B}}_{d}K\right)=Q+K^{T}RK, \end{array}$$

where *K* is the linear feedback gain designed to ensure that all eigenvalues of the closed-loop matrix lie strictly within the unit circle $\left|\mathrm{eig}\left(A_{d}-{\bar{B}}_{d}K\right)\right|<1$, thereby ensuring discrete-time stability. The operator eig(·) denotes the eigenvalues of a matrix.

The optimization problem of the controller is formulated as follows:

(25) $$\left\{\begin{array}{c} \arg\min_{v}J\left(k\right)\\ z(n|k)\in Z,\;\mathrm{for}\;n=1,2,\ldots ,N\\ v(n-1|k)\in V,\;\mathrm{for}\;n=1,2,\ldots ,N\\ z\left(N|k\right)\in Z_{f}\subset Z, \end{array}\right.$$

where Z and V are convex sets representing the admissible state and input constraints, respectively. The terminal set $Z_{f}$ satisfies $Z_{f}\subset X\subset Z$, where X is an invariant set for the closed-loop system near the origin.

### 3.3. Disturbance Observation and Adaptive Classification Mechanism

Conventional RMPCs rely on predefined disturbance bounds to tighten control and state constraints, which restricts control authority and limits maneuverability under disturbances. To address this issue, an adaptive input adjustment mechanism is proposed, leveraging real-time disturbance observation and grading to dynamically adjust control bounds. Accordingly, the disturbance observer is designed as follows:

(26) $$\left\{\begin{array}{cc} \dot{\lambda } & =-\kappa \lambda -\kappa \left(\Phi (V_{b},\Omega_{b},\tilde{\alpha },{\tilde{\alpha }}_{\delta },{\tilde{\alpha }}_{real},f,A,\gamma ,\beta )\right)\\ \hat{\Delta } & =\lambda +\kappa V_{b,x}, \end{array}\right.$$

where κ serves as the observer’s tuning gain, reflecting the sensitivity of the estimation dynamics to external disturbances and governing the convergence rate of the disturbance estimation, while λ represents the internal state variable that accumulates and filters disturbance information within the observer. The function Φ(·) can be expressed as a composite function, where $M_{ib}$ denotes inertial coupling terms arising from body–fluid interaction, $F_{fin}$ represents the hydrodynamic forces induced by fin deflections, $H_{b}$ accounts for nonlinear body hydrodynamics such as added mass and damping, and $T_{p}$ captures the periodic thrust fluctuations generated by the caudal fin oscillation. Together, these components characterize the primary sources of external and internal disturbances acting on the robotic dolphin. $\tilde{\alpha }$ denotes the velocity angle in the vertical plane, while $D_{\delta }$ and *D* are correction coefficients for the attack angle of the flipper and the main body. The final estimated disturbance $\hat{\Delta }$ incorporates both the observer state and the current surge velocity $V_{b,x}$.

To distinguish the direction of disturbances, a directional discrimination expression is further constructed based on the sequence of historical disturbance estimation $\hat{\Delta }\left(j\right)$ as follows:

(27) $$\begin{array}{c} \mathrm{sign}\left(w\left(k\right)\right)=\mathrm{sign}(\max\{|\hat{\Delta }\left(j\right)|,j=1,2,\ldots ,j_{\max}\}), \end{array}$$

where *j* is a positive integer, and its maximum value $j_{\max}$ is constrained by the control cycle and the flapping frequency of the robotic dolphin.

It is important to note that the performance of the disturbance observer is susceptible to modeling errors and high-frequency unmodeled dynamics, which can degrade estimation accuracy. Consequently, direct feedforward compensation may be impractical and may lead to undesirable oscillations or system instability. To address this, a disturbance-grading mechanism is introduced, where the disturbance intensity is smoothly mapped using a sigmoid function:

(28) $$\begin{array}{c} \mathrm{idx}=\mathrm{sigmoi}d_{1}\left(\max\{\right|\hat{\Delta }\left(j\right)|,j=1,2,\ldots ,j_{\max}\}). \end{array}$$

This design avoids directly applying disturbance estimates to control input updates, thereby effectively enhancing system stability. By tuning the parameters of the sigmoid function, the grading mechanism can be flexibly adjusted to meet control requirements under varying disturbance conditions.

### 3.4. Design of Control Allocation Strategy

As the robotic dolphin may operate in varying flow conditions, identical hydrodynamic forces and moments may require different control surface deflection angles. To address this, an improved adaptive control allocation strategy is proposed:

(29) $$\left\{\begin{array}{cc} J= & \min_{\delta_{p},\;\gamma }\left(q_{1}\delta_{p}^{2}+q_{2}\gamma^{2}\right)\\ s.t. & h\left(\mathrm{idx}\right)\cdot \left(k_{d}\delta_{p}+k_{g}\gamma \right)=u_{1}\\ & \left(k_{d}\delta_{p}\right)\cdot \left(k_{g}\gamma \right)\geq 0\\ & |\delta_{p}|\leq \mu ,\;|\gamma |\leq \lambda . \end{array}\right.$$

Here, $q_{1}$ and $q_{2}$ are weighting coefficients used to balance the relative contributions of the flipper and flukes in the process of control allocation. $k_{d}$ and $k_{g}$ are proportional factors reflecting the sensitivity to the required hydrodynamic moments. $\delta_{p}$ and γ denote the flipper deflection and fluke offset angles, respectively, while μ and λ represent their corresponding nonlinear actuation saturations. h(idx) is a disturbance-adaptive control gain that dynamically modulates the coupling between the flipper and flukes based on disturbance intensity. This strategy enables flexible adjustment of control surface participation according to disturbance severity, thereby achieving rational control allocation and enhancing both response efficiency and disturbance rejection.

## 4. Simulation and Analysis

In this section, a series of simulation experiments are conducted to evaluate the performance of the proposed control framework under varying environmental disturbances. The evaluation focuses on validating the feasibility of the optimized reference paths, ensuring the accuracy of the disturbance observation mechanism, and verifying the robustness of the path-following controller. Overall, these results demonstrate the effectiveness and practical applicability of the proposed approach in complex and disturbance-rich underwater environments.

### 4.1. Minimum-Snap-Based Path Optimization

**Figure 4:**
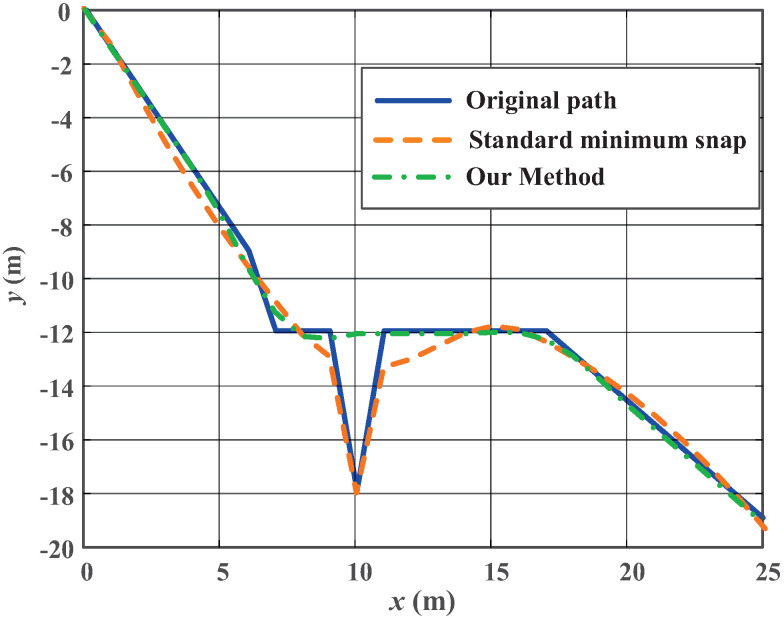
Path optimization aligned with the maneuverability characteristics of the robotic dolphin. The blue solid line denotes the original path, the orange dashed line represents the standard minimum-snap trajectory, and the green dash–dotted line indicates the trajectory optimized by the proposed method.

As shown in Figure 4, a series of path optimization experiments are conducted using the proposed curvature-constrained minimum-snap path optimizer to validate its capability in generating smooth and feasible trajectories for robotic dolphin path-following tasks. The solid line denotes a manually designed path, which includes a sharp segment at x=10 m. Throughout the path-following process, the robotic dolphin maintains a constant speed of 0.4 m/s. As illustrated by the dashed line, when applying the standard minimum-snap method, the resulting path exhibits significant deviations from the reference trajectory, especially in the segment with abrupt curvature changes. This deviation primarily stems from non-uniform time allocation across path segments, which induces excessive pitch angle fluctuations along the *y*-axis. These variations exceed the maneuvering limits of the robotic dolphin and consequently undermine the stability and safety of its path-following performance. In contrast, the path generated by the curvature-constrained method, as depicted by the dash–dot line, integrates a curvature constraint mechanism that accounts for the current speed and pitch limitations during optimization. This mechanism identifies and smooths infeasible path points that violate curvature constraints, thereby preventing sharp trajectory spikes and reducing abrupt attitude changes. These results demonstrate that the proposed optimization method effectively generates smooth paths that satisfy the dynamic constraints of the robotic dolphin. This method can be utilized as a pre-processing module within the planning system, providing a safe and robust trajectory basis for underwater path-following control.

### 4.2. Disturbance-Grading Performance and Robust Path-Following Control

**Figure 5:**
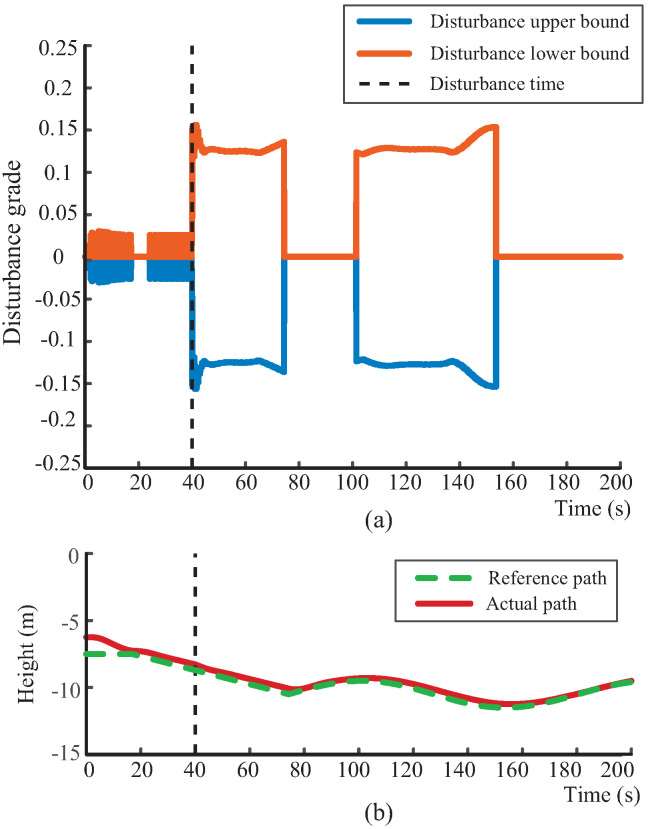
Performance validation of the disturbance-grading mechanism under a 0.04 m/s vertical flow. (**a**) Disturbance-grading output; (**b**) path-following response. The orange line shows the disturbance upper bound, the blue line the disturbance lower bound, the red line the actual path, and the green dashed line the reference path.

**Figure 6:**
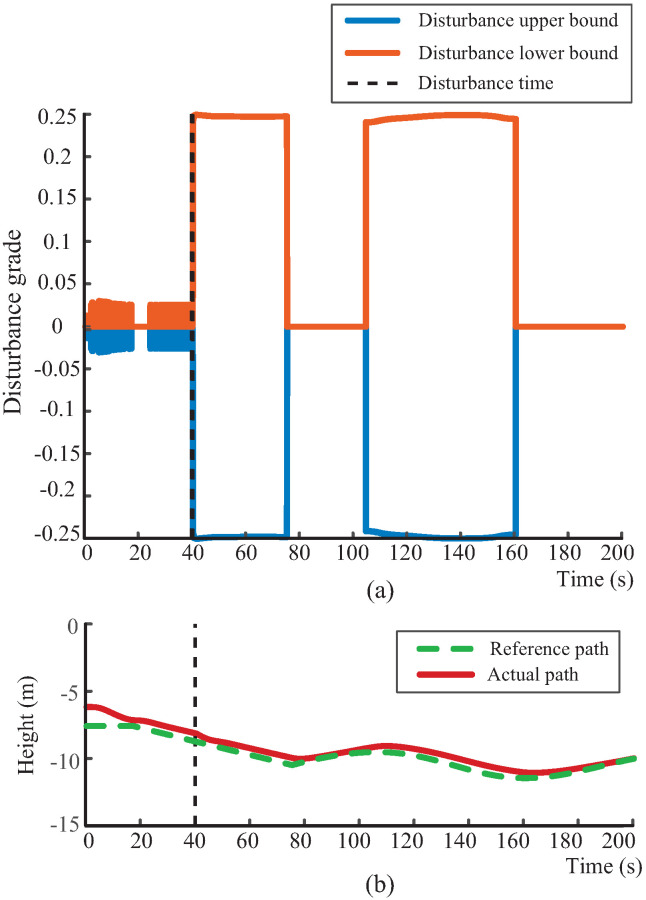
Performance validation of the disturbance-grading mechanism under a 0.08 m/s vertical flow. (**a**) Disturbance-grading output; (**b**) path-following response. The orange line shows the disturbance upper bound, the blue line the disturbance lower bound, the red line the actual path, and the green dashed line the reference path.

**Figure 7:**
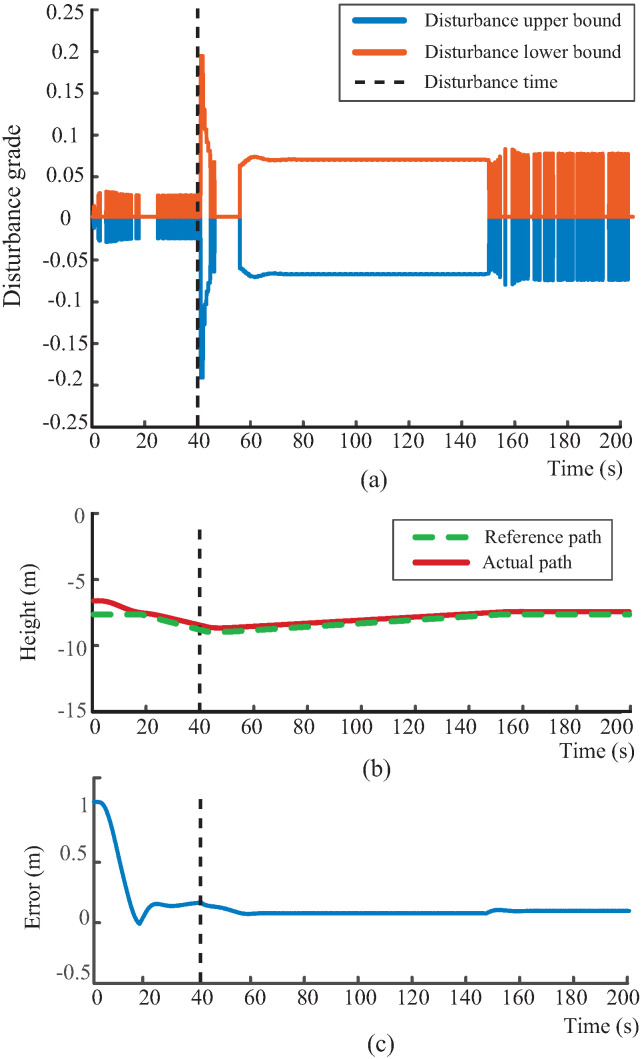
Path-following simulation under an environmental flow disturbance of [−0.5, 0.0, −0.04] m/s. (**a**) Disturbance-grading output; (**b**) Path-following performance; (**c**) tracking error. The orange line shows the disturbance upper bound, the blue line the disturbance lower bound, the red line the actual path, and the green dashed line the reference path.

To further validate the effectiveness of the proposed disturbance-grading mechanism and adaptive margin adjustment strategy, a series of simulations are conducted under various disturbance conditions. As shown in Figure 5a, Figure 6a, and Figure 7a, the proposed disturbance observer effectively identifies variations in fluid interference and produces the appropriate disturbance grade in response to an external perturbation introduced at t=40 s. Notably, it exhibits a distinct step response at the onset of the disturbance, highlighting its strong dynamic responsiveness. Moreover, the disturbance grade remains stable throughout the disturbance, confirming the effectiveness of the margin adjustment mechanism defined in (27). This mechanism adaptively adjusts the disturbance bounds based on the alignment between disturbance and path-following directions. When the environmental flow aligns with the diving direction of the robotic dolphin, the system releases the control margin for disturbance compensation, maintaining a stable disturbance grade. Conversely, when the directions are misaligned, the system relies on its propulsion and attitude regulation to actively counteract the disturbance. This indicates that combining disturbance observation with an adaptive grading mechanism forms a coordinated response framework that significantly enhances perception and compensation under external disturbances.

Furthermore, the adaptability and performance of the proposed robust path-following controller under varying disturbances are demonstrated in Figure 5b, Figure 6b, and Figure 7b. These results demonstrate that the proposed path-following method maintains robust tracking performance under various environmental disturbances, with the actual trajectory closely following the desired path. Additionally, the tracking error in Figure 7c further verifies the effectiveness of the proposed controller. Even under strong and multi-directional flow disturbances, the tracking error remains bounded and swiftly converges to near zero once the disturbances diminish, thereby supporting high-precision near-bottom path-following control. It is worth noting that, although the experiments are conducted under constant flows, the graded disturbance observer effectively decomposes time-varying disturbances into a sequence of stage-wise constant levels. Therefore, the validation under different constant flows also supports the applicability of the proposed method to dynamic disturbance conditions.

The simulation results clearly demonstrate the disturbance rejection capability and control stability of the proposed method under highly perturbed conditions, underscoring its strong potential for real-world applications. By integrating the disturbance-grading mechanism, adaptive control margin adjustment, and robust model predictive control, the method establishes a unified and resilient framework that ensures effective operation in complex and dynamic underwater environments. This framework enables the robotic dolphin to achieve high-precision path following and maintain stable performance, even under complex, time-varying, and unpredictable disturbance conditions.

## 5. Conclusions and Future Work

In this study, a robust path-following control method is proposed for a novel robotic dolphin to meet the operational demands of highly disturbed underwater environments. First, a novel robotic platform is developed by integrating a dorsoventral propulsion mechanism with a passive peduncle joint, and a full-state dynamic model tailored to the structure characteristics is established. To ensure dynamic feasibility and trajectory smoothness during path following, a minimum-snap-based path optimization method is proposed to generate reference paths that align with the maneuverability characteristics of the robotic dolphin. Subsequently, a robust model predictive controller is designed by incorporating a nonlinear disturbance observer and a disturbance-grading mechanism. Combined with state feedback and optimized control allocation, the controller enables a path-following framework with high dynamic responsiveness and strong disturbance rejection. Finally, extensive simulations and experiments are conducted to verify that the proposed framework maintains stable performance under varying flow disturbances, achieving an average path-following error below 0.09 m with a maximum deviation of 0.2 m. These results demonstrate that the proposed method enables stable and robust path-following control for the robotic dolphin, laying a solid foundation for future underwater operations in highly disturbed environments. Overall, the proposed approach is generalizable to various biomimetic robotic platforms and underwater tasks, offering a scalable solution for robust and adaptive control in complex aquatic environments.

Although the present experiments are conducted in simulation environments, future work will focus on validating the proposed framework under real-world conditions with nonlinear and turbulent disturbances, thereby providing stronger evidence of its robustness and engineering applicability. Additionally, multi-sensor data fusion and adaptive path-following control in three-dimensional terrain will be key directions for future research.

## Data Availability

The data generated during the current study are available from the corresponding author on reasonable request.
